# Evolution of Supramolecular Systems Towards Next-Generation Biosensors

**DOI:** 10.3389/fchem.2021.723111

**Published:** 2021-08-19

**Authors:** Sujeung Lim, Yuyao Kuang, Herdeline Ann M. Ardoña

**Affiliations:** ^1^ Department of Chemical and Biomolecular Engineering, Samueli School of Engineering, University of California, Irvine, Irvine, CA, United States; ^2^ Department of Biomedical Engineering, Samueli School of Engineering, University of California, Irvine, Irvine, CA, United States; ^3^ Department of Chemistry, School of Physical Sciences, University of California, Irvine, Irvine, CA, United States; ^4^ Sue & Bill Gross Stem Cell Research Center, University of California, Irvine, Irvine, CA, United States

**Keywords:** supramolecular, biosensors, self-assembly, host-guest interactions, supramolecular analytical chemistry

## Abstract

Supramolecular materials, which rely on dynamic non-covalent interactions, present a promising approach to advance the capabilities of currently available biosensors. The weak interactions between supramolecular monomers allow for adaptivity and responsiveness of supramolecular or self-assembling systems to external stimuli. In many cases, these characteristics improve the performance of recognition units, reporters, or signal transducers of biosensors. The facile methods for preparing supramolecular materials also allow for straightforward ways to combine them with other functional materials and create multicomponent sensors. To date, biosensors with supramolecular components are capable of not only detecting target analytes based on known ligand affinity or specific host-guest interactions, but can also be used for more complex structural detection such as chiral sensing. In this Review, we discuss the advancements in the area of biosensors, with a particular highlight on the designs of supramolecular materials employed in analytical applications over the years. We will first describe how different types of supramolecular components are currently used as recognition or reporter units for biosensors. The working mechanisms of detection and signal transduction by supramolecular systems will be presented, as well as the important hierarchical characteristics from the monomers to assemblies that contribute to selectivity and sensitivity. We will then examine how supramolecular materials are currently integrated in different types of biosensing platforms. Emerging trends and perspectives will be outlined, specifically for exploring new design and platforms that may bring supramolecular sensors a step closer towards practical use for multiplexed or differential sensing, higher throughput operations, real-time monitoring, reporting of biological function, as well as for environmental studies.

## Introduction

**FIGURE 1:**
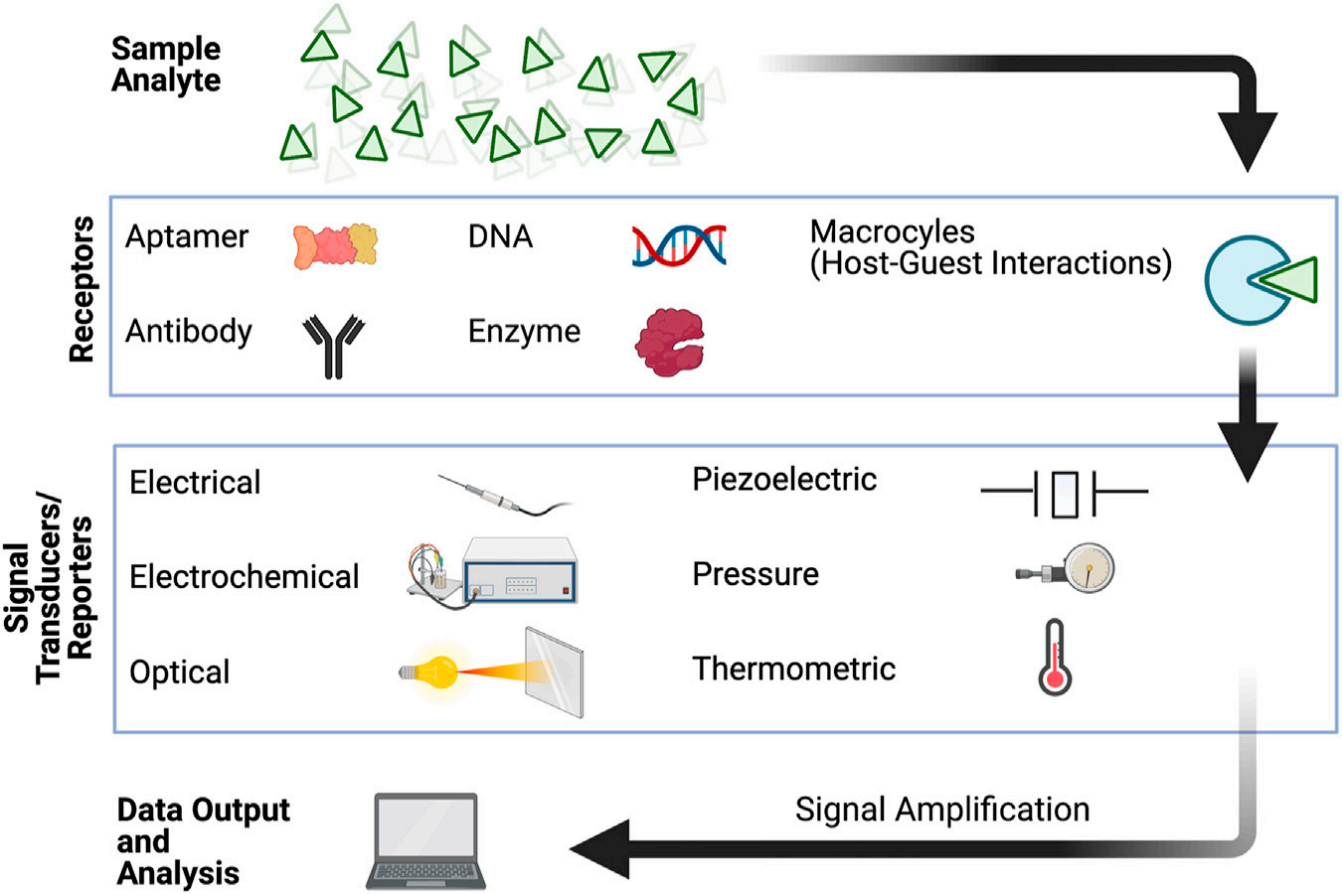
Schematic representation of the components involved in detection and signal transduction for biosensing.

The development of sensing platforms that can detect target analytes in biological milieu has since transformed the workflow in fields such as disease diagnostics, drug discovery, and food industry (Bhalla et al., 2016; Vigneshvar et al., 2016). These biosensors commonly rely on chemical, immunological, or enzymatic sensing elements whereby the kinetics and affinity of receptor-target binding at the molecular level are critical to their efficiency (Bhalla et al., 2016; Lim and Ahmed, 2017). There are currently several forms of biosensors that can successfully monitor biological analytes by reporting chemical, optical, electrical, or a combination of these signals (Figure 1). For example, biosensors are used not only to screen pathogens and prevent food contamination, but also, they are used to identify and detect the level of glucose, heart failure, and other diseases (Mehrotra, 2016). Since the development of the first biosensor in 1962 (Clark and Lyons, 1962), which was used for oxygen detection, the range of analytes that can be detected by these biosensors have now expanded from ionic species or small, neutral organic molecules, to cellular phenotypes (Mehrotra, 2016; Mako et al., 2019). Despite several advancements in the area of biosensing, currently available biosensors are reported to still have challenges associated with them, such as long-term stability, low sensitivity, selectivity at low target concentrations, and most importantly—the ability to perform under real-world environments.

**FIGURE 2:**
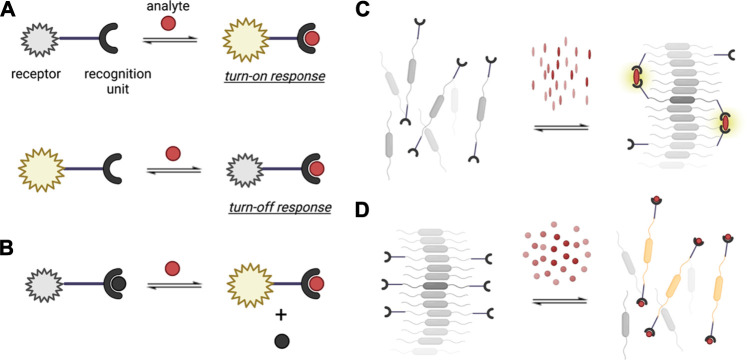
Different recognition mechanisms utilized in supramolecular analytical chemistry. **(A)** Direct sensing, illustrating a turn-on and turn-off response; **(B)** indicator displacement sensing; and sensing based on supramolecular **(C)** assembly/**(D)** disassembly.

More recently, supramolecular materials brought together by dynamic non-covalent interactions, such as host-guest interactions mediated by H-bonding, have been utilized as biosensing elements. The reversible nature of bonds that hold supramolecular monomers together provide several advantages for biosensing and for monitoring biologically-relevant analytes or signals in a continuous manner (Webber et al., 2016; Pinalli et al., 2018). Supramolecular analytical chemistry explores new design and properties of synthetic structures that can afford signal modulating molecular recognition and self-assembly processes *via* dynamic interactions (You et al., 2015). The non-covalent and adaptive nature of synthetic supramolecular units allow for multiple mechanisms of detection (Figure 2) that lead to increased signal-to-noise ratio and broadened range of analytes. Three general detection schemes, particularly for sensing systems that generate optical read-outs are the following: 1) direct sensing, whereby a signal output is generated upon the direct binding of an analyte to the receptor (Figure 2A); 2) indicator displacement, which involves the signal change upon the displacement of an indicator by an analyte from the sensory unit (Figure 2B); and 3) aggregation/disaggregation of sensory units in the presence of absence of the analyte (Figure 2C,D). The low energy barrier for disassembly and reassembly of supramolecular structure (Li J. et al., 2020), specifically those that are based on aggregates held by π-π interactions, also support good signal amplification. Curently available supramolecular materials have been made from inorganic systems, organic structures, polymers, hybrid materials, charged molecules, crystals, gels, metallic nanoparticles, and others by combining various types of non-covalent interactions (Martins et al., 2015; Wang et al., 2016). Many of these materials and their composites can be functionalized in a facile manner to achieve water solubility, making such supramolecular building blocks more relevant for sensing biological analytes under aqueous environments (Wang et al., 2016). Compared to top-down fabrication approaches such as etching and photolithography, the bottom-up fabrication of supramolecular materials allows the formation of biosensor elements with nanoscale dimensions (Nguyen et al., 2001; Aida et al., 2012; Kumar et al., 2018). Beyond harnessing unique signal transduction mechanisms from nanomaterials, the utility of supramolecular ensembles with nanoscale dimensions enables the miniaturization of biosensors which positively benefits the performance and applicability of biosensors. The higher surface area-to volume ratio increases the active sensing area, both enhancing the signal-to-noise-ratio and reducing the non-specific binding in biosensors (Adams et al., 2008; Soleymani and Li, 2017). Biosensors based on supramolecular ensembles also present higher local concentration of binding sites and lower interference from water molecules solvating the assemblies, resulting in highly sensitive recognition processes (Wang et al., 2016). Considering all of these properties, supramolecular materials are promising candidates for analytical applications and have the potential to address some existing challenges in the field of biosensors.

Herein, we will highlight key advancements in developing supramolecular systems for biosensing and use this as a roadmap to describe the next-generation of supramolecular biosensors. First, we will provide examples of supramolecular structure designs that serve as building blocks for biosensors operating *via* different signal transduction mechanisms. We will then feature representative examples of how certain supramolecular materials are used and implemented for various biosensing devices. To conclude, we will draw attention to emerging approaches for utilizing supramolecular systems, particularly how these may be adapted in the future towards better addressing the existing challenges in biosensing. The unique characteristics of supramolecular materials and the evolution in the design of their structures or device implementation will enable next-generation biosensors to measure a broader range of analytes, biological functionalities or responses with improved performance—towards positively contributing in environmental, pharmaceutical, and biomedical applications.

## Supramolecular Systems as Recognition and Reporter Units for Biosensing

### Macrocycles as Recognition Units

**FIGURE 3:**
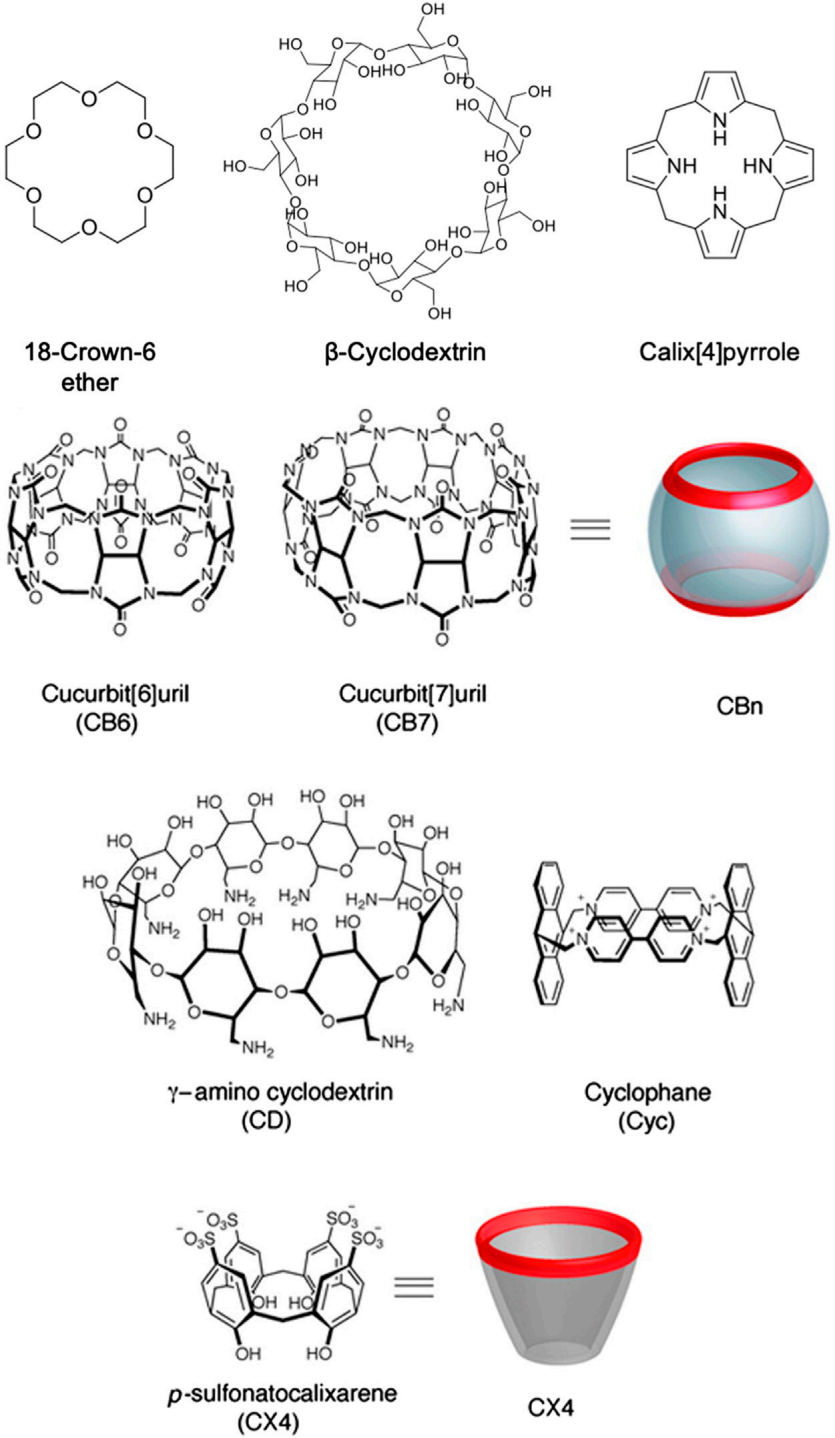
Examples of macrocycles used as supramolecular recognition units for biosensing. Adapted from Ghale et al., 2014. Copyright 2014 American Chemical Society.

Natural receptors, such as enzyme-substrate, protein-ligand, and antibody-antigen rely on non-covalent interactions, shape recognition, and binding site complementarities with high specificity. Several biosensors have employed these interactions to enhance selectivity (Kalantar-zadeh, 2013). In a similar fashion, synthetic supramolecular host-guest interactions, which typically involve macrocyclic systems, have been established as recognition elements in biosensors (Figure 3). For most macrocyclic hosts, the molecular recognition mechanism is based on the non-covalent entrapment of analytes as guest molecules in the host cavity. Macrocycles are considered to be chemically stable, easy to functionalize, and are suitable receptors for a wide range of analytes as guest molecules (Ghale and Nau, 2014; Pinalli et al., 2018). A variety of macrocycle functionalities can be achieved by the cyclization of different motifs based on aryl groups connected *via* short linkers—often resulting macrocycles with a hydrophobic inner part and hydrophilic outer part (Braegelman and Webber, 2019).

Among the most commonly used macrocyclic host is cyclodextrin (CD), which is synthesized through cyclization of glucose polysaccharides with α-1,4-linkage and can have tunable cavity sizes (Diehl et al., 2015; Wajs et al., 2016; Braegelman and Webber, 2019). The inner part of CDs is hydrophobic, whereas the outer part consists of hydroxyl moieties that facilitate water solubility. While adamantane-cyclodextrin is a widely used host-guest interaction for detection, CDs can also bind to a variety of nonpolar small molecule guests with binding affinities usually ranging from 100–1000 M^−1^ (Mako et al., 2019). Cucurbiturils (CB[n]s) is another class of water-soluble supramolecular host with a rigid cavity that can bind strongly with larger organic or metal cations (Diehl et al., 2015; Mako et al., 2019). CB[n]s are synthesized by the condensation of glycoluril and formaldehyde under acidic conditions, whereby the number of glycouril groups defines the size of the CB[n] cavity (Pinalli et al., 2018). The macrocycle rim of cucurbiturils are lined with carbonyls that result in a negative charge density at the cavity, which drives the binding of positively-charged guests along with solvation effects (Ling et al., 2016; Kaifer, 2018). Beyond sensing, cucurbiturils can also act as delivery vehicles for many hydrophobic drugs due to the hydrophobic nature of the host cavity (Diehl et al., 2015).

Another established class of macrocyclic supramolecular host is calix[n]arenes, which have been used as a receptor for both small cations and anions (Mako et al., 2019). Calixarenes can be formed by the condensation of a *p-*substituted phenol, resorcinol, or pyrogallol with an aldehyde. Calixarenes conjugated with naphthylidine have been reported to be capable of detecting amino acids such as cysteine, histidine, aspartic acid, and glutamic acid (Chinta et al., 2009). Hamuro and coworkers showed that calix[4]arenes could target a protein (cytochrome C) and inhibit the protein-protein interactions (Hamuro et al., 1997). Calixpyrroles, which are calixarene derivatives with conical conformation, have been demonstrated to bind to cations or anions depending on structural modification (Gale et al., 1996). Crown ether adds to this list of common macrocycles that can serve as a receptor for many metal ions chemical species, which is often incorporated in fluorescence-based sensor systems (Li et al., 2017). Unlike the other macrocycles discussed above, the binding affinities of neutral crown ethers for metal cations in organic solvents are generally stronger than in aqueous solution (Diehl et al., 2015). For example, 18-crown-6 binding of K^+^ has an association constant of 10^6^ M^−1^ methanol, whereas in water, it is only 10^2^ M^−1^ (Lamb et al., 1980). Preferential binding among different cations, such as between lithium vs. manganese cations, has also been demonstrated for crown ethers (Pauric et al., 2016).

Among the most recently explored class of macrocyclic host is pillar[n]arenes, composed of *p-*dialkoxybenzene-based repeating units connected by methylene bridges (Li Q. et al., 2020). Pillar[n]arenes can be synthesized *via* one-pot Friedel-Crafts alkylation of 1,4-dialkoxybenzenes, to form rigid macrocycles (Cai et al., 2021). They utilize ion-dipole interactions, solvophobic effects, and C-H-π interactions to interact with the guest compounds. These driving forces provide host-guest selectivity for different types of target analytes (Cai et al., 2021). Their electron-rich cavities can bind with cationic (e.g., toxic heavy metal ions) and neutral guests, but these cavities can also be functionalized to bind with anionic guests (Li Q. et al., 2020). For instance, Yin and co-workers have developed a sensor based on a water-soluble pillar[5]arene host and a planar chromophoric guest to detect Fe^3+^ ions with a 2.13 × 10^–7^ mol/L limit of detection (Yao et al., 2017). Apart from sensing and detection, pillar[n]arenes can also be used for targeted live cell imaging. Yao and co-workers have reported a supramolecular system with a two-step, sequential red fluorescence enhancement using pillar[5]arene-based host-guest recognition for mitochondria-targeted cell imaging (Guo et al., 2020). With this imaging construct, pillar[5]arene formed a complex with bicyanostilbene derivative (BSC8) due to the presence of two *N-*methylpyridin-1-ium groups. When pillar[5]arene/BSC8 complex was co-assembled with sodium dodecyl benzene sulfonate (SDBS), the red fluorescence was enhanced. In addition to pillar[n]arenes, prismarenes are also emerging macrocycles for supramolecular sensing. Gaeta and co-workers recently reported a templation-based thermodynamically controlled synthesis of primarenes, which have been demonstrated to have a good affinity for quaternary ammonium guests (Della Sala et al., 2020).

### π-Conjugated Assemblies as Reporter Systems

Supramolecular sensory ensembles with large π-systems or chromophores have emerged in recent years as effective reporter units for biosensing. These are often comprised of self-assembling π-systems with aggregation-induced changes in physical properties, such as absorption, fluorescence, or impedance, upon exposure to an analyte or other external triggers. For example, perylene-3,4:9,10-bis(dicarboximide) or perylene bisimide (PBI) is considered as an ideal fluorophore for sensors because it is an electron acceptor and exhibits strong fluorescence in its monomeric and small oligomeric states (Jones et al., 2004; Zhao et al., 2007). PBI and its analogues have been extensively studied as sensory units not only due to their excellent optoelectronic properties, but also for their stability under thermal and oxidative stress. The planar π-electron conjugation of PBIs allow for π-π stacking interactions amongst repeating units to form aggregates, resulting in fluorescence quenching and a hypsochromic shift of the absorption upon assembly (Zheng et al., 2005; Tang et al., 2007). PBI derivatives can be easily functionalized, which makes it more attractive for sensing with high specificity. In a recent example, the assembly system of a PBI derivative, *N,N*′-bis(2-(trimethylammonium)ethylene)perylene bisimide dichloride, was used as a reporter element for detecting biogenic amines based on electronic communication and effect of these amines on the aggregation (and therefore, photophysical properties) of dicationic PBI units (Bettini et al., 2019). Pyrene is another widely used chromophore for biosensors that has a large extinction coefficient, strong tendency towards π-π interactions, and good stability in aqueous solution when functionalized appropriately. The emission of pyrene is excimeric in nature, as characterized by a structureless fluorescence profile that is red-shifted by *ca.* 100 nm from the monomer emission (Wang et al., 2016). Charged pyrene derivatives are often designed to probe analytes that can electrostatically influence the aggregation of pyrene, which can be monitored by the increase or quenching of excimer emission. More examples of self-assembling π-systems for biosensing with optical readouts will be discussed in the subsequent sections.

Graphene is another interesting supramolecule that has caught attention for sensing applications in the recent years (Cho et al., 2020). While it is well known for its high mechanical strength, thermal conductivity, and elasticity (Pumera et al., 2010), the high surface-to-volume ratio of graphene enables the absorption of a large amount of aromatic biomolecules through π-π interactions (Geim and Novoselov, 2007), making it a favorable candidate for biosensing applications. Similarly, the high specfic surface area of graphene allows for direct contact with analytes resulting in high specificity and allows receptors to be efficiently immobilized on the graphene surface (Justino et al., 2017; Szunerits and Boukherroub, 2018). One limitation of graphene is that its native, unfunctionalized form has poor dispersion ability in aqueous medium. On the other hand, graphene oxide (GO) is easier to disperse and its nanoscale analogues have size-enabled properties that have already been leveraged for differential sensing of proteins, cells, and bacteria together with different fluorophores (Chou et al., 2012; Pei et al., 2012).

### Coordination Complexes as Reporter Units

Supramolecular coordination complexes (SCCs) with π-conjugated ligands, controllable coordination geometries, and tunable cavity architecture present several advantages for sensing applications (Cook et al., 2013; Liu et al., 2015; Dey and Haynes, 2021). Contrary to conventional fluorophores in small molecule probes, which experience signal quenching due analyte-induced aggregation, many SCCs display a higher signal-to-noise ratio due to aggregation-induced emission behavior in the presence of an analyte or trigger. The first wave of designs for these complexes were primarily designed for ion sensing. To date, 2D-metallacycles and 3D-metallcages have been used to probe larger ionic analytes, biomolecules, gases, and antibiotics based on changes in fluorescence emission intensities or quantum yields. Pt(II) complexes have been among the most widely used SCC for sensing applications. Recent examples include a Pt-based SCC capable of serving as a dual selective probe to detect both cations and anions, such as Zn^2+^ and pyrophosphate (Wong et al., 2021). Yam and co-workers demonstrated another recent example of an SCC involving Pt(II) complex used to detect RNA, RNA synthesis inhibitor, and nucleolus (Law et al., 2021). This guanidinium-functionalized alkynylplatinum(II) complex exhibited low cytotoxicity against HeLa and Chinese hamster ovary (CHO) cells, along with a low detection limit of 73.5 ng/ml for its luminescence-based sensing mechanism. Other examples of metal complexes have also now been used for chirality recognition of biomolecules (Folmer-Andersen et al., 2005; Folmer-Andersen et al., 2006; Dong et al., 2017; Mendez-Arroyo et al., 2017). Several efforts have also been dedicated to explore the influence of microenvironments on the sensing efficiency of SCCs as optical biosensors, which is important due to the heterogeneity of analyte environments for real-world applications. For example, Stang and co-workers demonstrated the effect of the number of metallacycle appendages and solvent polarity for SCC sensing (Tang et al., 2018). Their group reported other factors, such as the shape of coordination complexes, counter-anion, or substituent effects, that may influence the photophysical properties and sensing performance of SCCs (Yan et al., 2016; Zhou et al., 2016; Zhang M. et al., 2017). To explore the practical applicability of SCCs, Duan and co-workers reported SCCs with optical responses that can be utilized to detect amino acids even in human blood serum sample. They were able to show that their synthesized material (cerium-based tetrahedron with twelve hydrogen-bonding amide linkages and four triphenylamines) can selectively detect tryptophan/tryptophan-containing peptides in DMF-water mixtures (He et al., 2012)*.*

### Advancing Detection Schemes Using Nanoparticle Constructs

The sections above described general classes of compounds used for recognition and/or signal transduction. Under this section, presented are sensing elements that are specifically packaged to have structures within the nanoscale. This strategy often allows for enhanced signaling, multifunctionality, or better compatibility with bioimaging techniques due to improved systemic circulation dynamics. Nanoparticles for sensing that have been previously reported span the range of organic-inorganic composites, polymeric materials, and bioconjugated structures. Carbon-based nanoparticles, nanofibers, or nanotubes are widely used as nano-biosensors because they can be modified easily with functional groups and have high chemical stability. Carbon nanotubes (CNTs) are mainly used as electrochemical transducers in biosensors because of their large surface area, allowing an increase of immobilization of enzymes to the reaction area with high sensitivity and high electrical conductivity (Zhang et al., 2016; Cho et al., 2018). Similarly, the innate surface properties of CNTs allow biomolecules such as DNA and proteins to adsorb easily. CNTs can also be functionalized with hydrophilic units resulting in higher carrier capacity (Rao et al., 2011). Considering the relevance of these carbon-based nanomaterials for biological sensing, there have been several efforts focused on surface-functionalizing CNTs (e.g., with amino acids) or investigating the biodegradation of CNTs due to oxidative enzymes (Bianco et al., 2011; Tîlmaciu and Morris, 2015). A previous report showed CNT biodegradation due to human myeloperoxidase, demonstrating the potential of CNT-based DNA sensors for long-term biological use (Kagan et al., 2010). Fullerenes, which are considered as 0-D nanomaterials with low toxicity and good stability, have also been reported to be useful in electrochemical biosensors with low detection limits (Yuan et al., 2018; Wang et al., 2020).

Development of stimuli-responsive, amphiphilic nanomaterials or amphiphile-induced aggregation of nanoparticles have also emerged as a strategy to reduce concerns with instability under biological environments and toxicity. An early report from Heinze and co-workers demostrated an optical chemical/biochemical biosensor that is nanophase-separated due to amphiphilic polymeric networks (Hanko et al., 2006). This design allows for one phase to interact with the sensing elements, and another for the target analytes. Moroever, using amphiphilic sensors have high applicability for analytes that are also amphihilic in character such as glycolipids (Xu et al., 2018). Specific applications that have used amphiphilic structures for sensing include bacterial detection (Nandi et al., 2015) or assessment of pH-fluctuations in cancer cells or tumor tissues (Kim et al., 2020).

As a step towards developing sensing platforms for high throughput screening, nanoparticle array have also been developed for detecting biomacromolecule libraries. Rotello and co-workers have made huge strides on this front, particularly on using metal nanoparticle bioconjugates that utilize characteristic fingerprints for pattern recognition (Miranda et al., 2010b; Bunz and Rotello, 2010; Mout et al., 2012). For example, their group developed arrays of gold nanoparticle-fluorescent polymer complexes that were able to provide quantitative differentiation of multiple proteins with varying structural characteristics (Moyano et al., 2011). In another approach, enzyme-amplified array sensing (EAAS) was developed with gold nanoparticles, β-galactosidase as the enzyme, and an enzyme-activatable fluorescent probe (Miranda et al., 2010a). More recent versions of nanoparticle sensor arrays from Rotello and co-workers have been used to identify different mammalian cell types/states or to detect bacteria (Bajaj et al., 2010; Chen et al., 2015).

In the subsequent sections, we will be highlighting more examples of biosensors based on supramolecular systems, but with more emphasis on the mechanisms involved for signal transduction.

## Optical Supramolecular Biosensors

**FIGURE 4:**
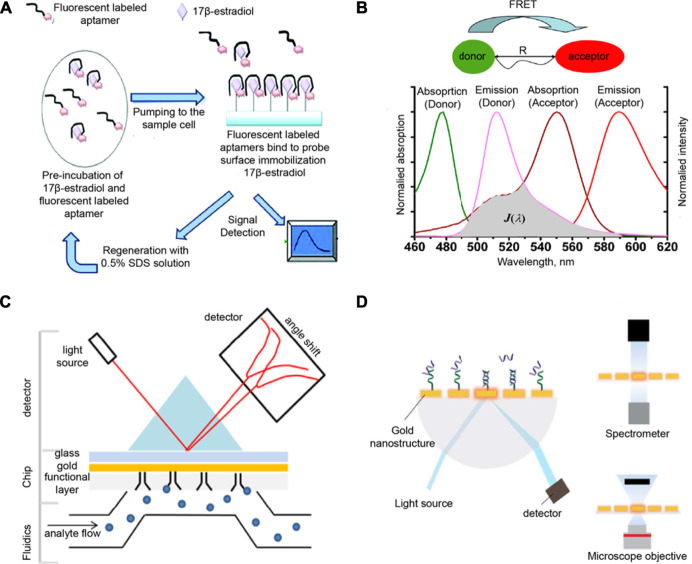
**(A)** Fluorescent labeling-based, **(B)** FRET-based, **(C)** SPR-based, and **(D)** LSPR-based mechanisms for optical biosensors. Adapted from (Yildirim et al., 2012; Yuan et al., 2013; Damborsky et al., 2016; Huertas et al., 2019). Copyright 2012, 2013 American Chemical Society; 2016 Portland Press.

Supramolecular biosensors with readouts based on changes in absorption, reflectance, emission, or interferometric pattern can operate under label-free or label-based sensing (Peltomaa et al., 2018). Regardless of the photonic process involved, optical biosensors are often considered to be highly sensitive, reproducible, and simple to use. The discussion below highlights a couple of optical processes utilized for biosensing (Figure 4).

Several optical sensors used for biologically relevant analytes utilize fluoresent probes, which either provide a “turn-on” or “turn-off” response. Sensors that use a “turn-on” response are deemed to be more generally favorable due to less background noise than a “turn-off” response. Early reports on supramolecular fluorescence sensors are largely based on chromophores that are able to change their emission upon binding of metal ions (Fabbrizzi and Poggi, 1995). For example, an anthracene-based supramolecular sensor with 3,8-bis-pyridin-4-ylethynyl [1,10]-phenanthroline (BPP) ligand was reported to be sensitive to micromolar concentrations of Ni^2+^, Cd^2+^, or Cr^3+^ (Resendiz et al., 2004). When BPP is self-assembled with 1,8-platinum-functionalized anthracene, the complex formed acts as a unit molecular clip which enables the optical sensing of transition metals. In a recent report by Gu and co-workers, a platform for highly selective detection of an endocrine disrupting compound (17β-estradiol) utilized a fluorescence-labeled DNA aptamer targeted for this analyte (Yildirim et al., 2012). Their portable, inexpensive, and reusable biosensor allows for real-time monitoring of 17β-estradiol through covalently immobilized recognition units onto the optical fiber sensor surface (Figure 4A).

**FIGURE 5:**
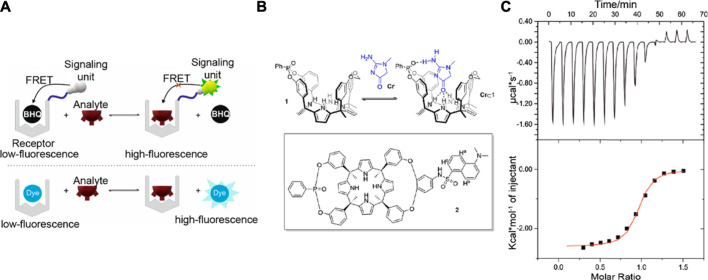
**(A)** Schematic showing the supramolecular fluorescence-based approach towards detecting creatinine. BHQ: a black-hole quencher **(B)** Top: Interaction of creatinine with a calix[4]pyrrole recognition unit. Bottom: Calix[4]pyrrole receptor is modified with dansyl fluorophore. **(C)** Top: Heat vs. time raw data for the calorimetry titration of the lipophilic creatinine derivative. Bottom: Normalized and integrated data from **(C)** top, showing the fit to the one-site binding model. Figures adapted from (Sierra et al., 2020). Copyright 2020 American Chemical Society.

Another widely used fluorescence-based phenomenon in biosensing is fluorescence resonance energy transfer (FRET), which occurs when energy transfer between donor and acceptor units occur as two interacting dipoles (Yuan et al., 2013; Mako et al., 2019; Wu et al., 2020a). FRET probes require the modulation of donor-acceptor distance or spectral overlap integral based on the analyte to be detected (Figure 4B). As an example, Wei et al. reported a metal ion FRET sensor that is highly selective towards potassium ion constructed using crown ether, carbon dots, and graphene (Wei et al., 2012). The dynamic carbon dots and 18-crown-6-ether-reduced graphene oxide hybrids (18C6E-rGO-Am-CD) complex were assembled. This platform can detect K^+^ concentrations relevant to the K^+^ content in the blood (3.5–5.3 mM). Moreover, this supramolecular sensor showed higher selectivity towards K^+^ as compared to other cations, which implies that the variation in the physiological concentrations of other ions have negligible effects on the read-outs. Sensitivity for other cations or even other biomolecules can be tuned for this sensor by combining different derivatives of crown ethers with carbon dots-reduced-graphene oxide. Additionally, the excitation (>450 nm) and emission (>500 nm) wavelengths of the sensing unit can minimize the background fluorescence from biological fluids. A more recent study by Nau and co-workers used a supramolecular FRET-based system for salmon sperm DNA sensing (Zhang et al., 2019). CB[7] was used as the recognition unit that was attached to a carboxyfluorescein (CF) dye as the FRET acceptor. The FRET donor is 4′,6-diamidino-2-phenylindole (DAPI), which can then intercalate with DNA. Upon increasing the DNA concentrations, DAPI moves farther from CB[7]-CF and does not serve as FRET donor. This relocation causes the fluorescnce intensity ratio to linearly increase in picomolar range (up to 20 μg/ml), with a limit of detection of *ca.* 60 ng/ml. This ratiometric, FRET-based sensing platform can be used as another method for DNA quantification with high sensitivity and reliability. Supramolecular biosensors with FRET probes have also been used for sensing metabolites such as creatinine. In the work by Sierra et al., calix[4]pyrrole phosphate-cavitands were used to sense creatinine and its lipophilic derivative hexylcreatinine (Figure 5; Sierra et al., 2020). They reported the use of calix[4]pyrrole modified with dansyl fluorophore to examine hexylcreatinine binding. The molecular recognition mechanism of the developed supramolecular biosensor for creatinine utilizes a combination of H-bonding, π-π, C-H-π interactions of polar groups of the receptor unit. Data from calorimetric titration revealed a one-site binding model for this system, as suggested by the sigmoidal binding isotherm with an inflection point around a host:guest ratio of 1:1. This sensor for creatinine is advantageous over other creatinine sensors because of the excellent binding ability to neutral polar species, mono- and polyatomic anions from cone conformation of the reporter unit. However, this reported supramolecular biosensor needs improvement in its selectivity to distinguish creatinine from other biologically relevant analytes such as proline and urea.

As mentioned in an earlier section, optical biosensors that depend on supramolecular assembly or aggregation of chromophores as triggered by the presence of analytes are also widely used. A fast, responsive humidity sensor reported by Mogera et al. used nanofibers built from self-assembled coronene tetracarboxylate (donor) and dodecyl methyl viologen (acceptor), which are photoactive components that can generate electrical readouts (Mogera et al., 2014). The response time of this sensor to relative humidity was reported to be only 10 milliseconds. This supramolecular sensing system is stable under ambient conditions and can even be stored up to 8 months. Other biosensors depend on conjugated aromatic compounds known as aggregation-induced emission luminogens (AIEgens), which often consist of flexible molecular moieties that can consume the energy of the excited state upon photoexcitation through intramolecular motion in the dispersed state. The fluorescence of AIEgens can be attributed to the restriction of intermolecular motion (Li J. et al., 2020). Supramolecular materials based on AIEgens could result in high luminescence efficiency and can be constructed easily to give controlled and tunable architectures (Li J. et al., 2020). Tang and co-workers reported the first AIEgen using tetraphenylethylene (TPE) as the supramolecular reporter unit (Hong et al., 2008). A more recent example from Lee an co-workers utilized a fluorescent “turn-on” peptide-modified TPE probe to detect heparin. This probe used electrostatic interactions and self-assembly to form supramolecular nanoparticles. The limit of detection of this sensor was 138.0 pM in water and 2.6 nM in serum sample (Lee et al., 2018). This sensor is the first example that shows the dual role of a fluorescent probe to detect and inhibit *via* the recognition process. Compared to the previously reported fluorescence methods for detecting oversulfated chondroitin sulfate, known as the heparin contaminant that can cause hypotension and angioedema, this fluorescence probe does not require a large amount of the enzyme and can be easily utilized for fast high-throughput screening.

Finally, under this section, we highlight surface plasmon resonance (SPR) as a widely utilized optical phenomenon for biosensors (Ogoshi and Harada, 2008; Dey and Goswami, 2011; Huertas et al., 2019; Chen and Wang, 2020). SPR allows for changes in refractive index to be measured in response to binding of analytes on the surface. This phenomenon occurs when the polarized light is reflected on the surface of metal at the interface of two media at a certain angle (Figure 4C). For example, Chen et al. used a hydrogel-gold nanoparticle supramolecular sphere to develop a label-free and real-time SPR imaging biosensor and specifically detect prostate cancer cell-derived exosomes (Chen et al., 2020). DNA probes on the gold chip surface modified with antibodies can capture the targets by forming polymers. Although the reported limit of detection for this biosensor is 1 × 10^5^ particles/mL, which is relatively inferior than the reported values of other nanomaterial-based methods for detecting exosomes, this sensor platform provided high response signals and also shows specificity against exosomes derived from different cells lines. This SPR imaging biosensor has the potential to be utilized in the clinical applications for early diagnosis and real time treatment monitoring of prostate cancer. On the other hand, localized SPR (LSPR) presents a modified SPR configuration that relies on distinct optical processes that occur due to the interaction of light with metallic nanostructures (Figure 4D). LSPR involves photoexcitation of metallic nanostructures that induces a collective electron charge oscillation and impacts the UV-visible absorbance (Damborsky et al., 2016). Sensing platforms based on LSPR offer a similar performance SPR systems without requiring high surface densities of recognition molecules.

## Supramolecular Sensing with Electrochemical and Electrical Read-Outs

**FIGURE 6:**
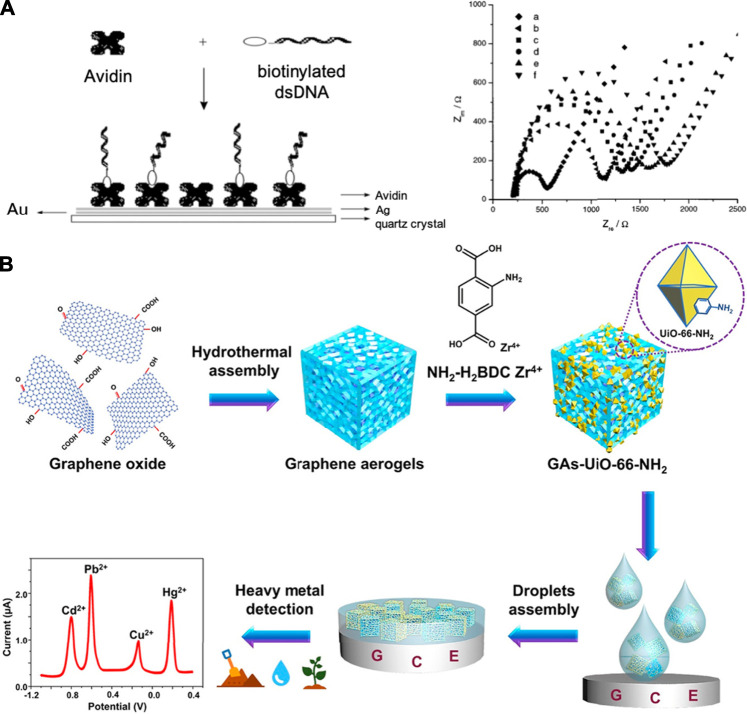
Examples of supramolecular biosensors with electrical readouts. **(A)** Sensor interface immobilized with double-stranded DNA onto electrodeposited avidin monolayer **(left)** and impedance measurements in the presence of Fe(CN_6_)^3-/4-^ using Au-based electrode with different surface modification **(right)**. **(B)** Electrochemical sensor based on a composite of graphene aerogel and metal-organic framework for simultaneous detection of multiple heavy-metal ions in aqueous solutions. Adapted from (Yan and Sadik, 2001; Lu et al., 2019). Copyright 2001, 2019 American Chemical Society.

Since the conception of the first electrochemical biosensor based on glucose oxidase, electrochemical read-outs have been predominantly used for biosensors, primarily due to their efficiency in metabolite monitoring (Chaubey and Malhotra, 2002; Turner, 2013; Hammond et al., 2016). Application of supramolecular materials in electrochemical biosensors not only improves the selectivity detection of biochemical reactions, but also increases the signal-to-noise ratio by minimizing the electrochemical sensor elements to nano-scale or micro-scale (Schoning and Poghossian, 2002). Electrochemical sensing in the presence of chemical and biological analytes typically involves electron transfer due to non-covalent interactions, which consequentially, alters the electrical properties of supramolecular systems in response to analyte exposure. This alteration can be converted to electrical signals and analyzed by various electrical read-out techniques such as potentiometry and amperometry (Grieshaber et al., 2008). Supramolecular systems that utilize electrochemical processes or electrical read-outs (Figure 6; Yan and Sadik, 2001; Lu et al., 2019) for analyte recognition has been widely used for ion quantification, protein sensing, nucleic acid analysis, and small molecule detection. To date, pushing the limit of detection to sub-nanomolar range has been a major driving factor for the development of potentiometric and amperometric biosensors (Bakker and Pretsch, 2005; Paul and Srivastava, 2018). One of the advantages of potentiometric biosensors is their independence from sample volume and biosensor size. This provides the potential to minimize the biosensor size and achieve high sensitivity at the same time (Ding and Qin, 2020). On the other hand, signal generation for supramolecular amperometric biosensors is based on charge-transfer processess that can produce measurable currents for the analysis. An amperometric biosensor offers advantages such as short response and analysis time, ease of use without sample pretreatment, broad detection range, and the possibility of miniaturization (Luo et al., 2018; Kawai et al., 2019; Takeda et al., 2021).

**FIGURE 7:**
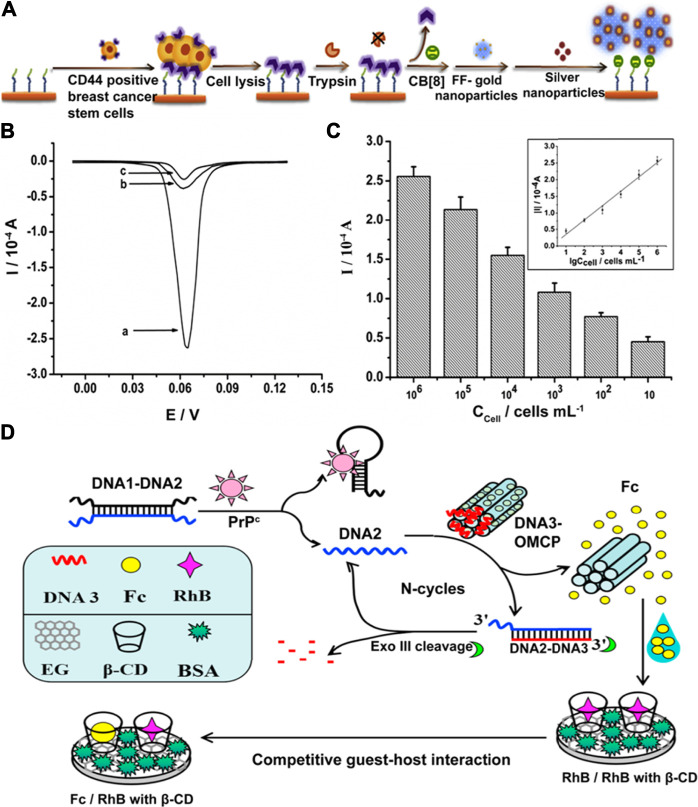
**(A)** Schematic illustration of detection of CD44 in cell samples. **(B)** Linear sweep voltammetry scans obtained in the presence of CD44-positive breast cancer stem cells (BCSCs) (curve a), CD44-negative cell BT474 (curve b) or in the absence of cell samples (curve c). **(C)** Peak currents obtained in different concentrations of BCSCs. Inset shows a linear relationship between the absolute value of the peak current and the logarithm of cell concentration from 10 cells/mL to 10^6^ cells/mL. Figures are adapted from (Zhao et al., 2018). **(D)** Schematic illustration of the label-free and cascaded dual-signaling amplified electrochemical strategy for cellular prion protein detection. Figure is adapted from (Yu et al., 2016b). Copyright 2016, 2018 Elsevier.

Healthcare diagnostics, such as the quantitative detection of disease-related proteins, is a major application of supramolecular electrochemical biosensors (Hewitt and Wilson, 2017; Merkx et al., 2019). As an alternative to PCR-based nucleic acid analysis techniques, an increasing number of studies apply electrochemical detection due to its rapid detection speed with high accuracy (Espy et al., 2006; Song et al., 2016; Fu et al., 2018; Drame et al., 2020). Zhao and co-workers developed a self-assembled supramolecular nanocomposite for the sensitive and selective electrochemical detection of CD44, an important surface biomarker of breast cancer stem cell (Zhao et al., 2018). Nanospheres self-assembled by diphenylalanine (FF) provide surfaces for gold and silver nanoparticle deposition to amplify the electrochemical signal. CB[8] links nanoparticles through host-guest interactions to aggregate at the electrode surface (Figure 7A). The recognition stability of this sensor is increased by using binding peptides as recognition units. Furthermore, the utility of gold and silver nanoparticles not only facilitates interactions between sensing elements, but also enable an ultra-high sensitivity for CD44 detection (Figure 7B,C). Aptamer-based electrochemical supramolecular biosensor is another promising platform due to its advantages, such as high sensitivity and fast response (Rivas et al., 2015; Yu et al., 2016a; Pereira et al., 2020). Yu et al. proposed a new strategy for using smart protein biogates in electrochemical detection of prion protein (PrP^C^) (Yu et al., 2015). The quantitative analysis of prion can be achieved on the basis of ratiometric electrochemical sensing using methylene blue (MB) and ferrocenecarboxylic acid (Fc). Protein biogates formed by the prion aptamer with MB strongly bind with β-CD on the electrode surface and prevent competitive binding by Fc. Taking advantage of only one single-labeled target aptamer enables a low detection limit (16 fM) and offers the potential for a facile, large-scale production of this biosensor. Later, the same research group proposed the first cascaded, dual-signaling, amplified electrochemical strategy for aptamer-based prion detection as shown in Figure 7D (Yu et al., 2016b). The free DNA2 released by specific and selective binding between PrP^C^ and DNA1 with PrP^C^-binding aptamer can hybridize with DNA3 to release the electroactive Fc from ordered mesoporous carbon probe (OMCP). This dual-signaling amplification can be achieved *via* the competitive guest-host interaction between Fc molecule/Rhodamine B (RhB) and β-CD for the high selectivity of detection. Recycling of DNA2 can be realized by dissociation through Exo III cleavage, which presents the advantage of being able to perform specific, repeatable, and robust assays using this sensor. Moreover, the application of enzymes for DNA recycling has the potential to be used in other DNA-based biosensors.

Reusability of sensors is another important design factor that has been realized with a couple of electrochemical biosensors. Yang and coworkers developed a pioneering example of a recyclable and immobilization-free electrochemical supramolecular biosensor for breast cancer early diagnostics (Yang et al., 2016). They reported a stable DNA sandwich structure formed by hybridization of MB-labeled signal DNA and alkylamino-modified capture DNA for a highly selective and ultra-sensitive detection of breast cancer susceptibility gene (BRCA). At the same time, host-guest interactions between this DNA sandwich structure with trithiocarbonate modified pillar[5]arene (P5A-CTA) allows for sensor regeneration by simple washing. This immobilization-free technology based on the host-guest interaction and homogeneous DNA hybridization has a high potential for practical applications due to its high reproducibility, ease of use, and reusability. Macrocycles such as β-CD and calixarenes have also been widely studied as a recognition element for nucleic acid electroanalysis due to good selectivity and fast readouts (Wang et al., 2005; Jiang et al., 2017; Barakat et al., 2020). For instance, supramolecular complex formed by ferrocenyl-β-CD and adamantylnaphthalene diimide not only yields strong electrochemical signals, but also stabilizes the whole target system due to threading intercalation with DNA strands (Sato et al., 2004). Furthermore, electrochemically active ferrocene is first masked by β-CD, and then released in the presence of target DNA. This allows for the analysis to be primarily based on the rise of an electrochemical signal instead of a drop, thereby operating under a “turn-on” detection scheme. In another example, Gorbatchuk et al. studied DNA damage using a copolymer of tetrasubstituted thiacalix[4]arene and oligolactic acid, then measuring changes on the polymer film properties such as permeability, charge distribution, and the charge transfer resistance (Gorbatchuk et al., 2017).

For the detection of biologically-relevant small molecules using supramolecular electrochemical sensors, signal specificity and binding selectivity are currently the most critical challenges for performance optimization. To determine two low-molecular weight tumor markers with similar structures, Shishkanova et al. reported a supramolecular receptor by functionalizing Troger’s base with amino- and coumarin-units to selectively bind with vanillylmandelic acid (VMA) in the presence of homovanillic acid (HVA) (Shishkanova et al., 2016). The spatial arrangement and accessibility of binding sites played a critical role in the selectivity of this biosensor. Control over receptor geometry provides a mechanism that utilizes spatial factor to enable high sensor specificity. In another example, Uppachai et al. applied supramolecular chemistry with surfactant assemblies to improve electrochemical sensor sensitivity for dopamine detection. The supramolecular assemblies formed by tetra-butylammonium bromide and sodium dodecyl sulphate enhance the electron transfer of dopamine due to hydrophobic interaction and electrostatic attraction between dopamine and gold nanoparticles on the modified glassy carbon electrode (Uppachai et al., 2020). Shen et al. reported a supramolecular aptamer self-assembled by the thiolated aptamer probe and a biotin-labeled analog in the presence of cocaine. This dual amplification resulting from the binding of linear DNA molecule and catalysis of the α-naphthylphosphate (α-NP) hydrolysis improves the limit of detection for cocaine to 1.3 nM (Shen et al., 2015). The mechanism of this rolling circle amplification is possible to be applied to the other detection of drug abuse in a fast and sensitive manner with suitable supramolecular aptamers.

**FIGURE 8:**
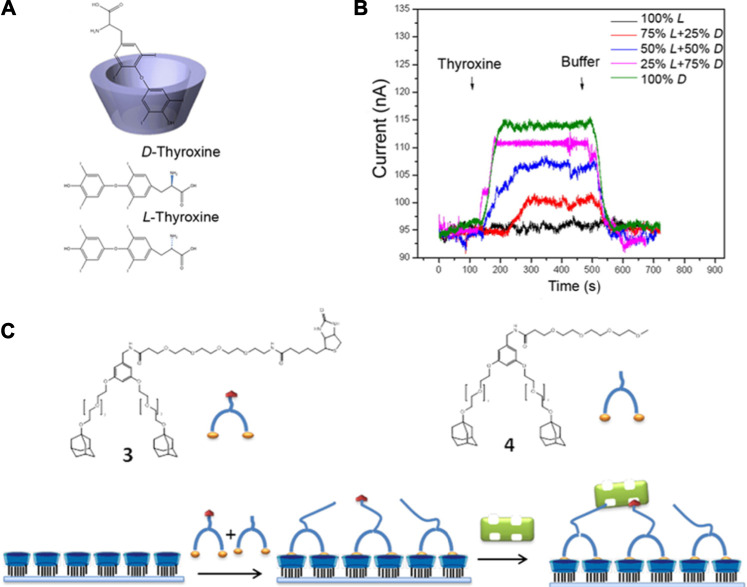
**(A, B)** Detection of 1 nM *L-* and *D*-thyroxine enantiomers using Si NWFETs functionalized with β-CD. **(C)** Adsorption scheme for the sensing of streptavidin through a mixture of adamantyl–biotin 3) and adamantyl–oligo(ethylene glycol) 4) at the surface. Modified from (Duan et al., 2013. Copyright 2013 American Chemical Society.

Lastly, it is important to highlight supramolecular sensing methods that rely on changes in electrical properties such as *via* impedance measurements and field effect transistors (FETs). Supramolecular sensing of enantiomeric composition using field-effect transistors (FET) has been reported with cyclodextrin-functionalized silicon nanowire FET (Si NWFET) (Duan et al., 2013). The supramolecular interface of this device was able to distinguish *L-* and *D-*enantiomers of thyroxine molecules (Figure 8). The reported affinity constants for *L*- and *D*-thyroxine are 1.02 ± 10^5^ M^−1^ and 7.11 ± 10^8^ M^−1^, respectively. The involved mechanism shed the light on supramolecular interface built by functionalization of Si NWFET with cyclodextrin to benefit both practical device design and fundamental research study. Another example of supramolecular FET-based sensing was demonstrated with CB[7] derivatives to detect amphetamine-type stimulants (ATS) (Jang et al., 2017). This OFET-based wireless sensoring platform offered a sensitive, flexible, and rapid approach for real-time liquid phase ATS detection. The limit of detection generated by this supramolecular biosensor was on the picomolar range, showing the highest sensitivity towards ATS to date. Moreover, the OFET based mechanism enables the feasibility to fabricate this portable and miniaturized sensor to drive the development of on-site real-time detection. Currently, there are several other macrocyclic receptors used in electrical sensors not only for solution-based analytes, but also for vapors. Calixarenes, porphyrins, and cyclodextrins are among those that have demonstrated good selectivity and fast readouts when coupled with electrical transducers for biosensing (Phillips et al., 2020).

## Other Transduction Mechanisms for Supramolecular Biosensors

While conventional biosensors that solely rely on photonic or electronic processes are widely used, there are other transduction mechanisms used for sensing which may offer unique advantages in terms of selectivity and sensitivity. These include, but are not limited to, piezoelectric, thermometric, magnetic, and micromechanical transducers. Discussed below are some examples of sensor systems based on supramolecular materials that utilize less common signal transduction mechanisms.

**FIGURE 9:**
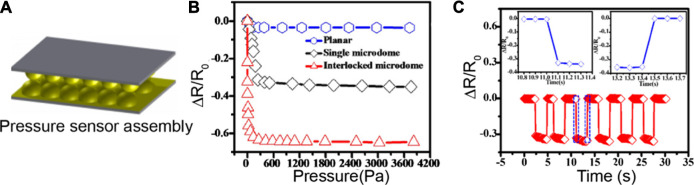
**(A)** Pressure sensor based on a flexible PDMS film, micropatterned using a colloid self-assembly technology. **(B)** The pressure sensitivities of sensor films with planar, and single microdome, and interlocked microdome surfaces. **(C)** Time-dependent response of the microdome-patterned PDMS sensor at a constant pressure of 100 Pa. Adapted from (Zhang Y. et al., 2017). Copyright 2017 American Chemical Society.

Biosensors that rely on pressure or force sensing do not require an analyte to bind to the sensors to initiate a readout. With this, pressure-based and piezoelectric biosensors have the potential to be used in portable devices for healthcare applications at home and point-of-care settings (Yu et al., 2021; Pohanka, 2018). In the report by Zhang et al., elastic microstructured polydimethylsiloxane (PDMS) film was constructed by using self-assembly method with monodispersed polystyrene (PS) microsphere as a monolayer (Figure 9A) (Zhang Y. et al., 2017). The surface microstructure, along with material durability and stability, were critical factors in determining the sensitivity of this supramolecular-based pressure sensor. This sensor exhibited a detection capacity at low pressures, affording a real-time change in resistance measurement by simply putting a dry rose flower on the top of the sensor. As the process of removing and placing the dry rose was repeated, the resistance values also increased and decreased, confirming the good limit of detection for this sensor. Moreover, by enhancing the size of the microdomes, the pressure sensors showed highly sensitive detection capability (∼15 kPa^−1^), fast repsonse time of 100 ms, and a low limit of detection of 4 Pa (Figures 9B,C). In addition, its tunability and facile fabrication process with low cost make this supramolecular pressure sensor potentially useful for real-time human health monitoring using wearable electronics. There have also been reports on supramolecular, stretchable pressure sensors such as the conductive self-healable gels (CSGs) by Khan and co-workers (Khan et al., 2020). This study reported a supramolecular gel, containing polythioctic acid (PTA), pyromellitic acid (PA), Fe^3+^ and a polyaniline (PANI) network, that is highly sensitive (2.8 kPa^−1^), stretchable (>5,000%), and has good strain sensitivity (gauge factor of 11). This supramolecular, self-healable gel sensor is injectable, making it an excellent candidate to be used in future biomedical applications. Quartz crystal resonators are also commonly used as piezoelectric biosensors because of the linear relationship that can be established between deposited mass and frequency response of the crystal standing wave (Pohanka, 2018; Chalklen et al., 2020). In a report by Liu et al., a piezoelectric supramolecular sensor was coated with β-cyclodextrin and calixarene derivatives to show high sensitivity and selectivity toward aliphatic amines. The frequency data demonstrates the sensitivity of this piezoelectric quartz crystal sensor to the size and the shape of the aliphatic amine analytes (Liu et al., 2002). In this case, the microstructural change of the host molecule dictates how sensitively the piezoelectric quartz crystal sensor coated with cyclodextrin derivatives detects the amine guests. For the piezoelectric quartz crystal sensors coated with calixarene derivatives, there are two main sensing mechanisms—formation of complex inside (*endo*) and outside (*exo*) the macrocycle—depending on the interaction with the amine guests. Further illustrating the advantages of a piezoelectric transducer, CB[6] was used for a sensor that can rapidly detect cocaine with high sensitivity (Menezes et al., 2017). This piezoelectric sensing platform also offers reusability for detecting drugs. These representative examples suggest that macrocyclic receptors can be systematically tuned to impart selectivity on piezoelectric systems for rapid sensing.

**FIGURE 10:**
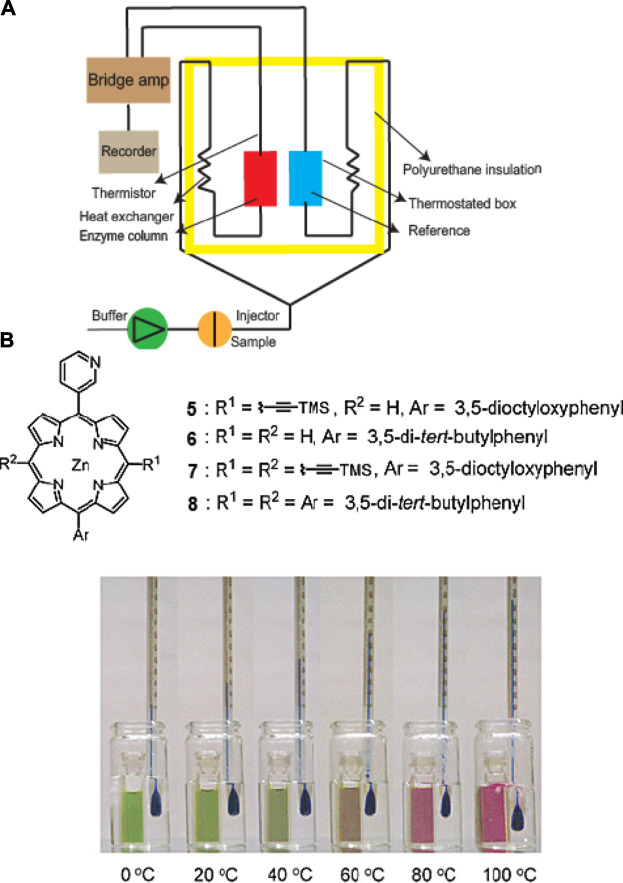
**(A)** Schematic diagram of a thermal biosensor. **(B)** Thermochromism exhibited by a zinc complex of alkynyl-functionalized (3-pyridyl)porphyrin in toluene. Adapted from (Tsuda et al., 2003; Zhou et al., 2012). Copyright 2003 American Chemical Society.

On the other hand, thermal biosensors offer the advantage of long-term stability since there is often no chemical contact needed between transducer and sample. A general workflow for a thermometric biosensor is shown in Figure 10A, which specifically illustrates an enzyme thermistor (Zhou et al., 2012). The thermostated box can regulate the physiological temperature, whereby the heat generated reduces thermistor resistance and the bridge amplifier reads the signal (Zheng et al., 2006; Zhou et al., 2012). A pioneering study on a supramolecular thermochromic system utilized a zinc-porphyrin complex with a metal-ligating 3-pyridyl group (Tsuda et al., 2003). This sensor takes advantage of the ability of the complex to have a thermally-induced change in axial coordination dynamics, ultimately leading to an altered absorption profile (Figure 10B). The temperature-dependent molecular transformation leads to a change in effective π-electronic conjugation length, resulting in absorption spectral shifts that are highly dependent on alkynyl group. In particular, one of the conditions led the zinc complex in toluene to change its color from green to yellow to red as the temperature increased. Without any alkynyl group, the color only slightly changed from orange to pink and having two alkynyl groups did not result in a dramatic color change as the temperature increased from 0 to 100°C. This reported sensor presents an example of wide-range thermochromism, thus, has the potential to be used as a multicolor thermometer with easy visualization of the colors representing corresponding temperatures. Another example of a supramolecular temparature sensor is reported by Sambe et al., whereby the sensor was constructed based on the host-guest interactions with hydrophilic tetracationic macrocyclic host cyclobis(paraquat-*p*-phenylene) tetrachloride (CBPQT^4+^) with a programmable functionality (Sambe et al., 2014). Finally, as an example of a sensor array system that utilizes a hybrid approach for signal read-outs, Zhang and co-workers developed a thermochemiluminescence (TCL)-based platform for protein and cell discrimination. The fingerprint TCL signals are uniquely generated as a function of thermal catalytic oxidation (Kong et al., 2011). The reported cross-reactive sensing array system, which can be categorized under a class of vapor-based sensors known as “chemical noses,” is composed of nanomaterials that are solid catalysts with good stability. This TCL-based sensing system is advantageous over other types due to enhanced sensitivity, reversibility, and fast generation of read-outs.

## Emerging Trends and Current Challenges

Our discussion up to this point has demonstrated the rich variety of exemplar molecular designs and signal transduction mechanisms used for supramolecular biosensors to date. These previously reported supramolecular sensors have afforded the detection of charged species, not limited to metal cations and anions, but also organics species such as amino acids. Beyond broadening the scope of target analytes, it is logical that the next steps in the field would be to explore ways that could increase the sensitivity, selectivity, and stability of supramolecular materials for biosensing. Optimization of these characteristics have been among the longstanding challenges that are relevant towards the practical applications of supramolecular sensors for biological imaging (Reineck and Gibson, 2017), technologies for national security (Sun et al., 2015), or food industry (Zhou et al., 2018)—to name a few. On a similar note, the rise of newer device platforms that enable real-time sensing and high throughput screening (e.g., microfluidics or flexible sensors) requires compatibility and stability of supramolecular structures present in real biological environments. Among the pioneering examples of microfluidic integration is the supramolecular optical chemosensor by Nocera and co-workers, which uses cyclodextrin modified with a Tb^3+^ macrocycle to detect polyaromatic hydrocarbons in aqueous solutions at sub-micromolar concentrations even without signal amplification (Rudzinski et al., 2002). Recently, gel-based supramolecular sensors have been emerging as a platform that provides a unique kinetics and dynamics for the sensing process (Cao et al., 2019; Ma et al., 2019; Sebastian and Prasad, 2020). For example, supramolecular copper metallogel has been used for sensing toxic cyanide ions (Sebastian and Prasad, 2020). This work presented cyanide sensing based on deprotonation in aqueous medium caused by very high solvation energy of cyanide ion in water. Beyond these aforementioned themes, discussed below are other promising trajectories that have been emerging in the field of supramolecular biosensors.

First, we are currently in an era where molecular machines (rotaxanes, catenanes, or molecular rotors) exist and continue to be explored for analytical applications. These complex structures offer the advantage of having fast-motion response that is fatigue-resistant, which may be used to detect submolecular movement (Erbas-Cakmak et al., 2015). Interlocked structures of molecular machines are advantageous for optically sensing small guest molecules. In a study by Cornell and co-workers, a biosensor with a self-assembled lipid bilayer embedded with gramicidin A ion channels has been developed to act as a biological switch (Moradi-Monfared et al., 2012). This rapid and sensitive diagnostic device, which preserves its accuracy even in the presence of human serum, plasma and whole blood, can be used as an alternative for enzyme-linked immunosorbent assay (ELISA) that does not require pre- or post-processing steps. A recent work from Stoddart and co-workers demonstrates the use of electrochemically switchable bistable [2]rotaxane with a fluorescent molecular rotor, which may be used in the future for electro-optical sensing applications (Wu Y. et al., 2020). Cyclodextrin-based catenanes and rotaxanes have also been reported for use in sensing many cations and anions (Bąk et al., 2020). For example, it was reported that rotaxane was used to sense Au^3+^ using fluorophores like anthracene (Chan et al., 2019).

New approaches towards differential sensing or multiplexing using supramolecular materials have also gained attention over the past decade. Sensor arrays that mimic the human sensory system (i.e., artificial nose or tongue) have been used to discriminate between multiple analytes. For example, Eker et al., reported a supramolecular luminescent sensor platform with five parallel sensing self-assembled monolayers incorporated in a microfluidic device that can detect multiple analytes (phosphate anions and aromatic carboxylic acids) (Eker et al., 2011). Other examples that involve supramolecular arrays for differentially sensing biomolecular analytes used antibody-free systems for the detection of the histone code (Minaker et al., 2012) and gold nanoclusters for nucleotide sensing (Rana et al., 2012; Pezzato et al., 2013), and even mammalian cell types or cancer states (Bajaj et al., 2010).

Since many biologically relevant molecules are chiral, and different enantiomers have varying biological activity or functionality, methods for chirality analysis are of high significance for biological analyte sensing. Supramolecular materials used for chiral sensing commonly utilize optical sensing mechanisms. Wolf and co-workers made several advances in this area, primarily by using achiral chromophores that exhibit induced circular dichroism and only show strong Cotton effects in the UV-Vis region upon addition of the chiral analyte. Several metal complexes have also been used for circular dichroism-based sensing. Additionally, combining circular dichroism readouts with SPR and FET sensors have been previously reported (Ariga et al., 2010). A recent report on chiral supramolecular biocoordination polymers with photochromic, photoluminescent, photoconductive, and chemiresistive characteristics present a future for chiral sensing whereby one system may afford multiple signal transduction mechanisms (Shang et al., 2018).

Next, advancements in platform engineering is required to achieve sensors that are portable, inexpensive, and can be used multiple times over. Reusability or recyclability of sensors is a well-sought property since many available sensors are based upon irreversible interactions. The supramolecular-based sensors achieve high stability during the recycling due to the unique interactions between sensor elements and analytes. For instance, Qu et al., developed a reusable supramolecular sensing platform using β-CD as a host to detect bacteria and proteins. They showed that it could be used to detect bacteria (*E. coli*) and protein (Concanavalin A) several times without losing bioactivity (Qu et al., 2017a). Another example is from Lu et al., which involves a silver nanocluster-based pH sensor together with a copolymer ligand that has *N-*heterocyclic groups of 8-hydroxyquinoline and *N-*isopropylacrylamide (Lu et al., 2018). This reversible sensing system was able to be reused to sense a pH range of 3.04–5.25 up to six times. Sonication or rinsing the sensor surface with a solution at a specific pH (Duan et al., 2015), surfactant addition (Tang et al., 2016; Qu et al., 2017b), or utilization of competitive binding (Jia et al., 2016; Lei et al., 2016) are other strategies that have been reported to effectively regenerate supramolecular sensing units.

Lastly, interfacing supramolecular sensors directly with living system—whether for mammalian cell screening or detection of bacteria or virus—is an area that continuously receives high interest in biosensing. This has been proven to be challenging due to the complex and dynamic microenvironments within or surrounding these target living systems. Nonetheless, the examples of supramolecular materials used for differentially screening or identifying cells (e.g., tumorigenic vs. healthy) and bacteria highlighted in earlier sections suggest that the field is now significantly past establishing the proof-of-principle for detecting these analytes. Recent studies that explore the supramolecular assembly dynamics within cellular environments or sub-cellular localization present the future potential of having more controlled detection schemes within the biological millieu (Krivitsky et al., 2019; Bai et al., 2020; He et al., 2020; Pieszka et al., 2020).

## Outlook

**FIGURE 11:**
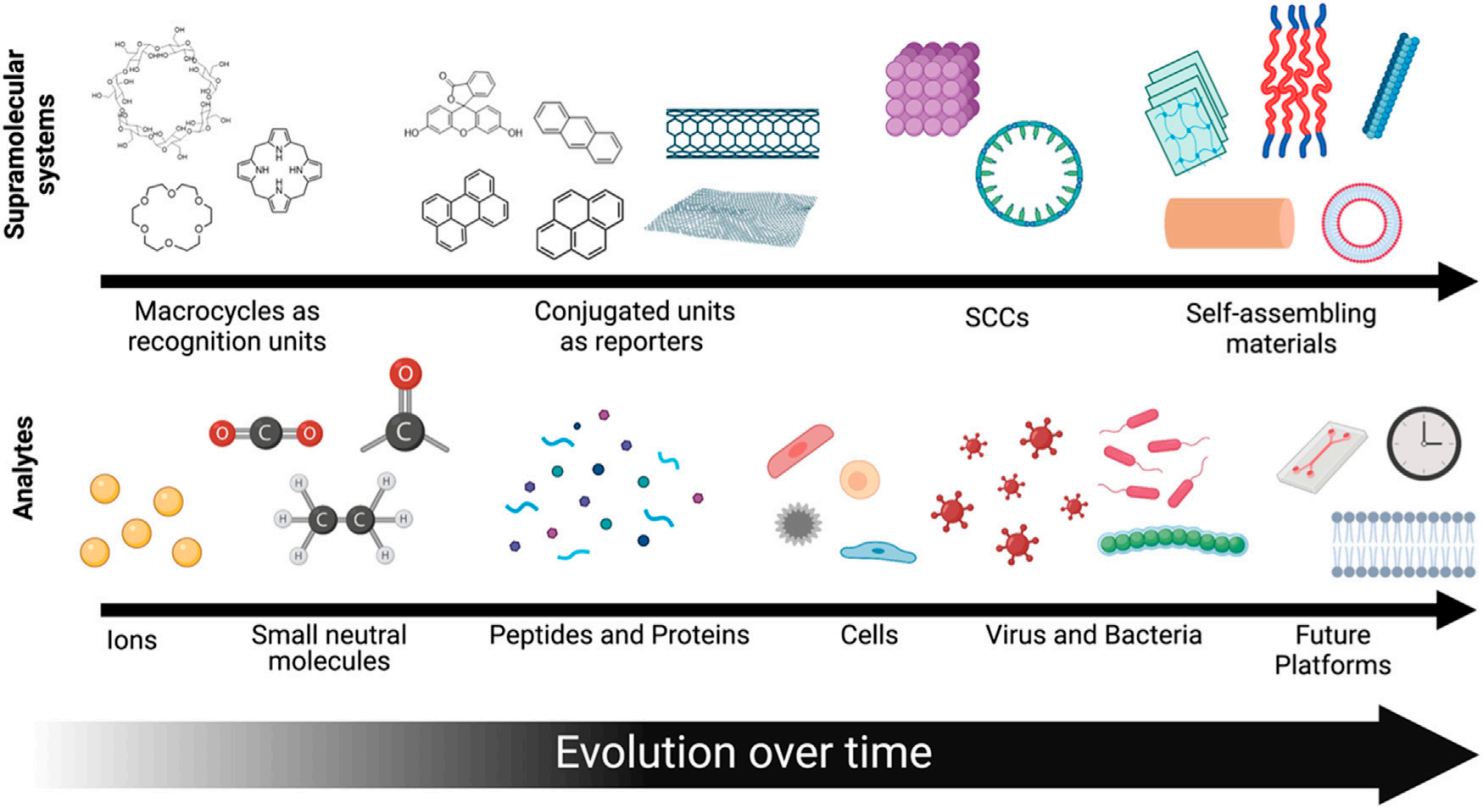
A schematic depiction of the evolution of supramolecular systems developed over time and the analytes that they can detect.

In this Review, we summarized the key technological advancements in the area of supramolecular biosensors (Figure 11). We have highlighted the evolution of utility of supramolecular components in biosensing platforms, from recognition units, to now being able to be both detectors and transducers. Advancements in supramolecular analytical chemistry have led to innovative designs for high performing biosensors that have transformed the types of analytes and biological niches where these sensors can be applied—from small ionic species, to now being able differetially detect proteins, cell phenotypes, and bacteria. The analytical supramolecular systems developed to date are getting closer to truly mimicking the natural sensory systems of higher order species, relying on selective interactions with a broad range of analytes instead of a specific interaction with just one type of analyte. As such, beyond the single analyte sensing paradigm, supramolecular sensors can now report environmental parameters (such as temperature and pressure), differentiate between stereoisomers, and perform multiplexed sensing. While this review is not meant to provide a comprehensive history of every iteration of supramolecular sensor design reported to date, we hope to have highlighted the key advancements that has led to the state-of-the-art supramolecular biosensors nowadays.

Due to the innumerable variations of supramolecular structures that may be designed and synthesized, one can expect that the next-generation of supramolecular structures can open doors for more complex recognition and transduction functionalities that are yet to be realized. Such advancements can bring supramolecular-based biosensors a step closer towards its practical use for real-time drug delivery monitoring, reporting of tissue function of 3D organ models, or as commercial components of wearable health monitors. Overall, supramolecular biosensing has evolved over the years into a rich, transdisciplinary field with high promise towards more practical applications in future technologies.
